# Engaging Rural and Racial-Ethnic Populations in Gerontological Research

**DOI:** 10.1093/geroni/igab046.1237

**Published:** 2021-12-17

**Authors:** Ishan Williams

**Affiliations:** University of Virginia, Charlottesville, Virginia, United States

## Abstract

Older adults from racial/ethnic populations, as well as rural-dwelling older adults, are often at heightened risk for experiencing health disparities. Reasons for these disparities may include access issues, language barriers, distrust, lack of awareness, and of culturally appropriate materials. Racial/ethnic populations and rural-dwelling populations are also less likely to be included in research to help minimize the impact of these disparities. Shifting from reducing disparities to eliminating disparities will require attentiveness to designing programs and research that focus on increasing representation of racial/ethnic groups in research, integrating diverse populations (particularly rural and other marginalized groups) into the development of ideas and projects, and finally a commitment to culturally appropriate and inclusive approaches to research and education. Applying these strategies can provide guidance on how to best facilitate inclusive and equitable research, collaborative partnerships, and equitable healthcare for everyone, especially those from populations often underrepresented.

